# Selective modulation of chemical and electrical synapses of *Helix* neuronal networks during in vitro development

**DOI:** 10.1186/1471-2202-14-22

**Published:** 2013-02-25

**Authors:** Paolo Massobrio, Carlo NG Giachello, Mirella Ghirardi, Sergio Martinoia

**Affiliations:** 1 Neuroengineering and Bio-nano Technology Group (NBT), Department of Informatics, Bioengineering, Robotics, System Engineering (DIBRIS), University of Genova, Genova, Italy; 2 Department of Neuroscience, University of Torino, Torino, Italy; 3 Istituto Nazionale di Neuroscienze, Torino, Italy

**Keywords:** *Helix* neurons, Micro-Electrode Arrays, Functional connectivity, Network development, Dynamics

## Abstract

**Background:**

A large number of invertebrate models, including the snail *Helix*, emerged as particularly suitable tools for investigating the formation of synapses and the specificity of neuronal connectivity. *Helix* neurons can be individually identified and isolated in cell culture, showing well-conserved size, position, biophysical properties, synaptic connections, and physiological functions. Although we previously showed the potential usefulness of *Helix* polysynaptic circuits, a full characterization of synaptic connectivity and its dynamics during network development has not been performed.

**Results:**

In this paper, we systematically investigated the in vitro formation of polysynaptic circuits, among *Helix* B2 and the serotonergic C1 neurons, from a morphological and functional point of view. Since these cells are generally silent in culture, networks were chemically stimulated with either high extracellular potassium concentrations or, alternatively, serotonin. Potassium induced a transient depolarization of all neurons. On the other hand, we found prolonged firing activity, selectively maintained following the first serotonin application. Statistical analysis revealed no significant changes in neuronal dynamics during network development. Moreover, we demonstrated that the cell-selective effect of serotonin was also responsible for short-lasting alterations in C1 excitability, without long-term rebounds.

Estimation of the functional connections by means of cross-correlation analysis revealed that networks under elevated KCl concentrations exhibited strongly correlated signals with short latencies (about 5 ms), typical of electrically coupled cells. Conversely, neurons treated with serotonin were weakly connected with longer latencies (exceeding 20 ms) between the interacting neurons. Finally, we clearly demonstrated that these two types of correlations (in terms of strength/latency) were effectively related to the presence of electrical or chemical connections, by comparing Micro-Electrode Array (MEA) signal traces with intracellularly recorded cell pairs.

**Conclusions:**

Networks treated with either potassium or serotonin were predominantly interconnected through electrical or chemical connections, respectively. Furthermore, B2 response and short-term increase in C1 excitability induced by serotonin is sufficient to trigger spontaneous activity with chemical connections, an important requisite for long-term maintenance of firing activity.

## Background

In the past, the relative simplicity of the nervous system of invertebrates such as *Helix* snails allowed the detailed study of many aspects of neuronal connectivity [1-3], neurite outgrowth [4], synapse formation and plasticity [4-7] by using an in vitro approach (reviewed in [8]). In this experimental preparation, a large number of neurons can be individually identified and isolated in cell culture. The study of reciprocal interactions among identified neurons in small reconstructed circuits was pioneered in 1990 by Kleinfeld et al. [9] by using *Aplysia* neurons. More recently, the connectivity between C1, C3, and B2 *Helix* neurons was first investigated at the level of micro-networks (i.e., pairs of synaptically connected neurons, whose activity is recorded by means of intracellular sharp electrode techniques) [5], and later at the level of large-networks [10], by exploiting the technology offered by Micro-Electrode Arrays (MEAs). In vivo, the serotonergic C1 neurons, localized in the ventral side of cerebral ganglia, are synaptically connected to B2 neurons in the buccal ganglia [11], and they are involved in the regulation of feeding behaviors of *Helix* snails. In this way, it is feasible to reconstruct in dissociated cell culture synaptic connections among individually identified invertebrate neurons that resemble in vitro their in vivo features [4]. *Helix* neurons are large in size (soma diameter up to 100–150 μm) compared with the microelectrode diameter (30 μm) of MEAs. This geometrical ratio makes possible a 1:1 coupling between neurons and microelectrodes (not easy to do with mammalian neurons), facilitating the study of the relationship between the electrophysiological activity of individual neurons in a network, as well as the development of neurite outgrowth. However, the aforementioned studies were acutely performed, recording the electrophysiological activity for a few hours once the circuit has been established.

To the best of our knowledge, in vitro studies about long lasting (i.e., days) recordings of invertebrate neuronal networks cannot be found. The possibility to couple *Helix* neurons to MEAs, allows recordings of the electrophysiological activity and monitoring the development of network organization can modulate such activity in the long term.

Indeed, MEA technology is the “gold standard” to study the development of in vitro neuronal networks, as demonstrated by many studies related to mammalian cortical networks [12-16]: during development, while synapses and neural connectivity build up, neuronal network activity starts to self-organize [17], and modulates its electrophysiological patterns (i.e., spike and bursts).

Following this approach, the purpose of this work is to study and characterize the dynamics and connectivity of *Helix* networks made up of C1 and B2 neurons coupled to MEAs during their development.

*Helix* neurons start to create synaptic connections just a few hours after plating, and their development is faster (couple of days) than cortical cultures from mammals (3–4 weeks in vitro) [13]. A peculiarity of these invertebrate neuronal networks is the absence of spontaneous activity: the *Helix* neurons used in this study were found to be generally silent on MEAs [10,18], and spontaneous firing was observed only occasionally. Therefore, we triggered neuronal activity by means of chemical treatments, namely potassium chloride (KCl) which induces general depolarization of the cell membrane potential and serotonin (5-HT) which selectively depolarizes B2 neurons. Thus, the induced activity was followed during development at fixed time-points (i.e., 6, 24, 30, 48, 54, 72 hours after cell plating) and analyzed by means of the first-order statistics used to characterize neuronal dynamics: inter spike interval (ISI) and firing rate. The estimation of the functional connections established among neurons of the network was made by using cross-correlation function [19] in order to reconstruct the topological connections, and monitor them during development. Considering the simplicity of these neuronal circuits, a comparison with the morphological development of the network was possible, and a good matching between morphological and functional links was achieved.

We found that both chemical stimulations (i.e., KCl and 5-HT) were efficacious to trigger firing activity in *Helix* circuits, but a long-lasting change in activity occurred only with 5-HT treatment. We did not observe a modulation of the dynamics as far as the network evolved, although we counted an increase with time of functional connections. Moreover, the analysis of spiking activity as well as functional link latencies suggest that networks treated with 5-HT displayed a dynamic modulated mostly by chemical synapses, while a predominance of electrical connections occurred in KCl-triggered networks.

## Results

Experimental data collected by MEAs and presented in this work were obtained from 8 *Helix* cultures, monitored at the 6, 24, 36, 48, 54, and 72 hours after plating. As explained in the Methods Section, we considered 3 cultures treated with serotonin (5-HT), 2 with extracellular high-potassium (KCl), and 3 as control, i.e., without any kind of treatment.

### *Helix* polysynaptic circuits display a treatment-dependent long-lasting activity

**Figure 1:**
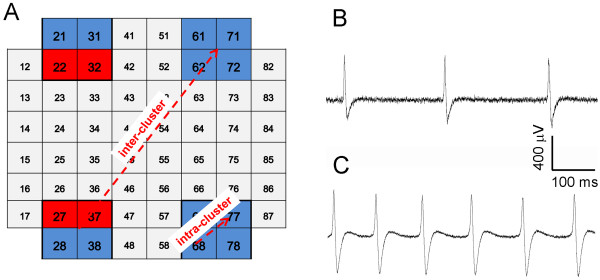
**Schematic representation of the network organization and raw data.** (**A**) Sample scheme of neuron placement onto a MEA. Red and blue squares indicate the position of C1 and B2 cells, respectively. The number inside the squares indicate the electrode position in the MEA layout. (**B**) 1 second of activity from a microelectrode with a C1 neuron. (**C**) 1 second of activity coming from a microelectrode with a B2 neuron.

When *Helix* neurons are cultured in vitro, they do not display any kind of electrical spontaneous activity [10]. We followed the development (from 6 to 72 hours after plating) of 3 cultures made up of 12 B2 and 4 C1 neurons arranged in the configuration depicted in Figure 1A. At each time-point, we recorded 30 min of spontaneous activity: no spike activity was observed in the 3 non stimulated cultures. To verify the healthy conditions of the networks, and the effective presence of synaptic connections, we delivered electrical stimulation at the 72^nd^ hour. Selected cells were singularly impaled and stimulated with a current pulse whose amplitude was sufficient to overcome the excitability threshold. Under these conditions, we were able to observe a network activity which involved all neurons of the network (data not shown).

To trigger electrophysiological activity in *Helix* networks, we chemically treated such cultures by means of two stimulation protocols at each time-point of recording: i) Increasing extracellular potassium concentration (KCl) from 5 mM to 65 mM, that results in a general depolarization of the membrane potentials of cells; ii) delivery of serotonin (5-HT) in a concentration equals to 20 μM that selectively depolarizes B2 neurons. The details of these protocols are described in the Methods Section.

**Figure 2:**
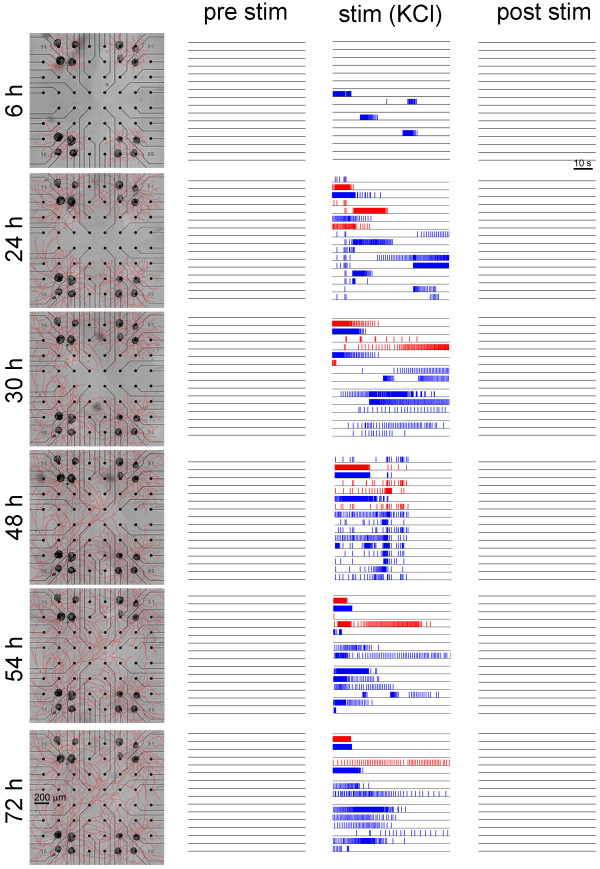
**Example of *Helix *culture manipulated with KCl applications.** Each block represents a time-point of recordings (i.e., 6, 24, 36, 48, 54, 72 hours after plating). In the first column, the development of the neurite arborizations is indicated (scale bar is 200 μm). The three columns of raster plots show one minute of electrophysiological activity just before, during and after the KCl treatment. Red and blue lines indicate C1 and B2 neurons respectively. Scale bar is 10 s.

**Figure 3:**
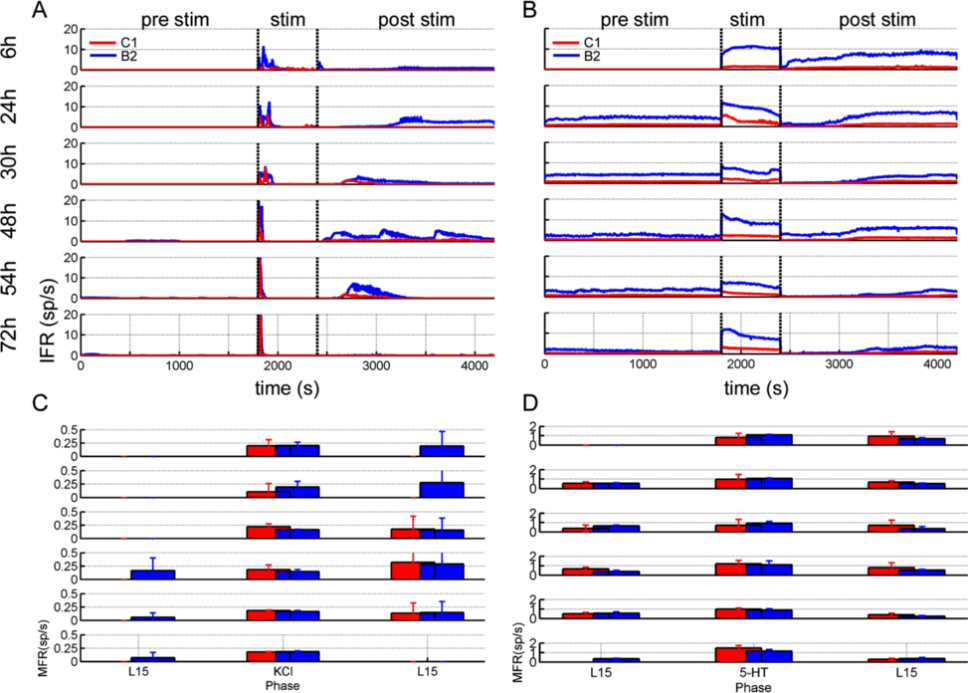
**Firing characterization of *Helix *networks.** Instantaneous firing rate (IFR) (**A**, **B**) and Mean firing rate (MFR) (**C**, **D**) averaged over the entire dataset for the cultures treated with KCl (A, C) and 5-HT (B, D). C1 and B2 neurons are coded with red and blue colors, respectively.

Figure 2 shows the qualitative results from one culture treated with KCl. Each horizontal block of the figure represents a recording time-point (i.e., 6, 24, 36, 48, 54, 72 hours). The three raster plots per each block show one minute of electrophysiological activity before, during and after the KCl delivery. Red and blue lines are for C1 and B2 neurons, respectively. The first observation is that KCl induced a transient depolarization of the network which lasted no more than 1 minute: neurons quickly depolarized with a delay of 825 ± 43 ms from the KCl delivery. As a result of the sustained membrane depolarization, networks moved toward a quiescent state before the wash-out, possibly through the inactivation of voltage-gated sodium channels. The phases before and after the KCl stimulation were mainly characterized by the absence of activity: before the stimulation, *Helix* networks were always completely silent (cf., also the IFR graph reported in Figure 3A). After the wash-out the network reached a resting state, where no activity was recorded: only little oscillations in the B2 neurons were occasionally found, as shown in Figure 3A. The main observation from these raster plots and from the IFR curves from 24–72 hours of development is that KCl does not induce persistent activity in the network. This transient behavior was constant for all the periods of recording during development.

**Figure 4:**
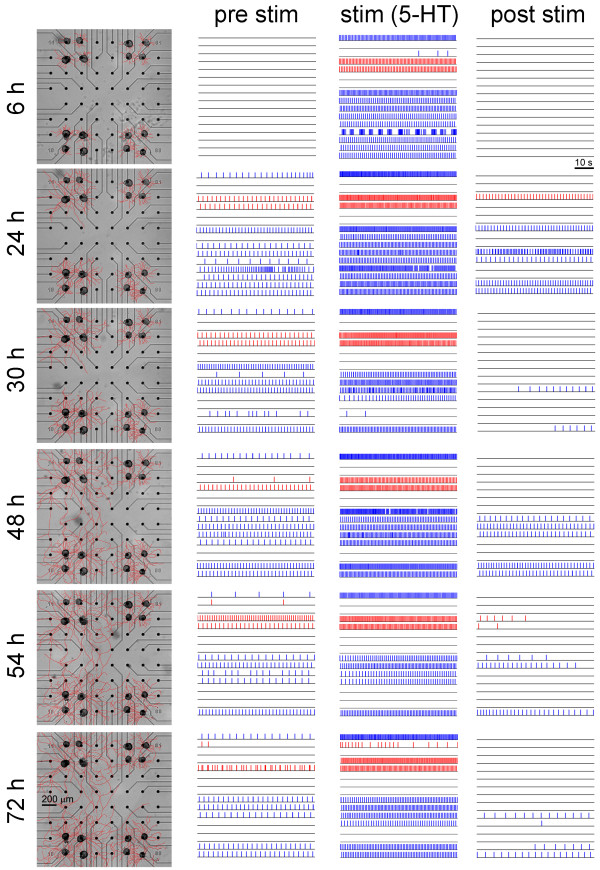
**Example of *Helix *culture manipulated with 5-HT applications.** Each line represents a time-point of recordings (i.e., 6, 24, 36, 48, 54, 72 hours after plating). In the first column, the development of the neurite arborizations is indicated (scale bar is 200 μm). The three columns of raster plots as in Figure 2. Red and blue lines indicate C1 and B2 neurons respectively. Scale bar is 10 s.

Conversely, a long-lasting neural activity in networks treated with 5-HT were observed. Figure 4, arranged with the same graphical layout of Figure 2, shows an example of *Helix* network treated with 5-HT. Except for the raster plots of the first block (first and third column, i.e., before and after the 5-HT stimulation, respectively), neurons continued to fire after the wash-out (third column), and several hours later (first column). The delivery of 5-HT seems to trigger the neuronal network circuits towards an oscillatory state which persists for long-term during network development. We estimated the frequency of the quasi-periodic behavior of the C1 and B2 cells by computing the reciprocal of the ISI. Once entered into a periodic regime, we found that C1 neurons fired at a frequency which range from 0.2 Hz during the pre-stimulation phase, to 0.6 Hz during the stimulation and then decreasing to 0.3 Hz in the post stimulus phase. These values were maintained during the network development. B2 neurons exhibited a firing frequency of about 1 Hz during the periodic regime. To assess whether the firing activity was periodic, we computed the coefficient of variation (CV) of the ISI. By setting an arbitrary threshold at 0.2, we considered quasi-periodic those neurons which presented a CV less than 0.2. Additional file 1 shows the trends of both the CV and the frequency. The networks treated with KCl never displayed a quasi-periodic behavior, since CV was always greater than 0.2.

To better analyze the mechanisms underlying 5-HT-induced long-lasting activity, we further characterized *Helix* networks in order to evaluate whether this event is related to changes in cell excitability or, alternatively, to modifications of morphological and functional connectivity.

### *Helix* networks show stable dynamics during development

**Figure 5:**
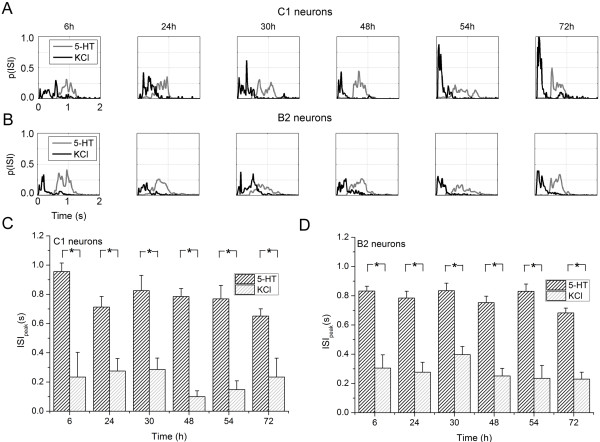
**Inter spike interval (ISI) characterization of *Helix *networks.** (**A**, **B**) ISI distribution relative to the stimulation phases for (**A**) C1 and (**B**) B2 neurons. The treatment with 5-HT and KCl is coded with gray and black colors, respectively. Bin width = 20 ms. (**C**, **D**) Temporal peak position of the ISI distributions relative to C1 (**C**) and B2 (**D**) neurons, respectively. (Kruskal-Wallis, non-parametric test, *P* < 0.01)

Following the approach used to characterize the in vitro development of mammalian cortical neurons coupled to MEAs [12,13], we first quantified the mean firing rate (MFR) and the inter spike interval (ISI). Figure 3A and B shows the IFR trends of the C1 (red) and B2 (blue) neurons. By comparing the IFR trends of the cultures treated with KCl (Figure 3A) and 5-HT (Figure 3B), the main difference was the total absence of electrophysiological activity in both C1 and B2 neurons before the KCl stimulation. On the contrary 5-HT induced a low-frequency (< 5 Hz) baseline activity, especially in the B2 cells. During development, no remarkable differences were noticed: in cultures stimulated with KCl, we observed a rapid increase of activity immediately after the stimulation, which lasted less than 1 minute. Both in C1 and B2 neurons, the IFR reached a maximum value of 20 Hz. In the case of 5-HT stimulation the IFR after a sudden increase remained stable through all the stimulation phase. No main differences were observed during development. C1 and B2 neurons fired in a very regular way: B2 neurons fired at a higher frequency than C1, and this activity remained until the wash-out. Afterwards, after a brief transient (from 2 to 5 minutes) characterized by a silent period, B2 neurons spontaneously restarted to fire. In this post stimulus phase, the dynamics exhibited by the networks treated with KCl was more irregular: C1 neurons were always quiescent during development, whereas B2 ones showed a peak of activity at the 48^th^ hour. Then, the IFR slowed down at 54^th^ hour to zero at the 72^nd^ hour.

By quantifying the amount of spikes generated by networks stimulated by KCl or 5-HT during development, we computed the MFR as depicted in Figures 3C and 3D. Averaged over a time window of 30 minutes, the MFR peaks for the *Helix* neuronal networks treated with KCl, and 5-HT reached values of 0.4 and 1.5 sp/s, respectively. In particular, we found a maximum value of the MFR for the network treated with 5-HT equals to 1.16 ± 0.16 (B2 neurons) and 1.48 ± 0.27 (C1 neurons) sp/s during the stimulation phase at the 72^nd^ hour time-point. For the KCl manipulation, a peak in the firing activity of 0.29 ± 0.41 (B2 neurons) and 0.32 ± 0.45 (C1 neurons) sp/s was reached after 48 hours from the plating in the post stimulation phase. Interestingly, MFR values were statistically equal during development (Figures 3C and D), suggesting that spiking activity was stable although the network connectivity changed and evolved (cf., Figures 2, 4, and 7).

To better quantify possible changes in the network dynamics during in vitro development, we derived the inter spike interval (ISI) distribution as shown in Figure 5. Figures 5A and B show the ISI distributions relative to the stimulation phases (i.e., 5-HT (gray), KCl (black)) for C1 and B2 neurons, respectively. We found that KCl induced a faster spiking activity than 5-HT: both C1 (Figure 5A) and B2 (Figure 5B) neurons when stimulated with KCl (black line) presented a main ISI peak less than 0.5 s, while the 5-HT treatment (gray line) shifted such peaks for both C1 and B2 neurons towards 1 s. The latency of the main peak (preferred inter-spike interval) showed no statistically significant differences during development both for C1 and B2 neurons and for both stimulations (cf. Figure 5C and D). Thus, comparing the cultures treated with KCl and 5-HT, we found a statistically significant difference in the ISI temporal peak position (Kruskal-Wallis, non-parametric test, *P* < 0.01).

### Synaptically isolated *Helix* neurons do not show excitability changes with 5-HT and KCl treatments

**Figure 6:**
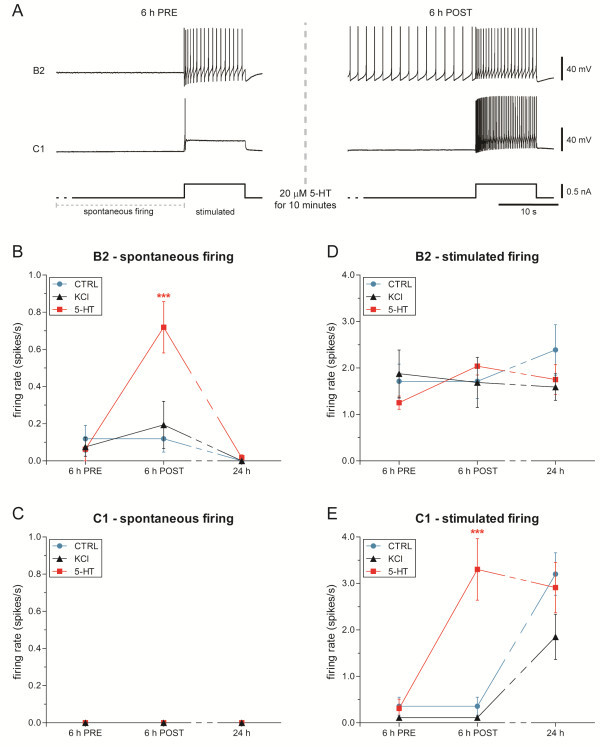
**Estimation of *Helix *C1 and B2 excitability.** (**A**) Representative electrophysiological recordings from C1 and B2 neurons before and after 5-HT application. Both spontaneous activity (**B**, **C**) and the response to a depolarizing current step (0.5 nA for 10 seconds; **D**, **E**) were measured. (**B**) B2 neurons showed a significant increase of spontaneous firing activity only after 5-HT treatment. At 24 hours after plating no activity was observed. Conversely, C1 neurons (**C**) were silent in all the experimental groups. During the electrical stimulation, B2 exhibited a similar firing rate (**D**), while a higher firing frequency was evoked in C1 neurons only after 5-HT application. (**E**) Comparable values were recorded 24 hours after plating.

In order to evaluate 5-HT or KCl-induced alterations in neuronal excitability, we measured biophysical parameters on cultured isolated C1 and B2 neurons, by using intracellular recording techniques. The same cell culture procedures and treatments described for MEA devices were applied to these cells. We analyzed both spontaneous and firing activity induced by a depolarization stimulus applied at 6 hours after plating, before the treatment and soon after the treatment washout (Figure 6A). Moreover, the same cells were recorded again at 24 hours after plating to check the presence of long-term effects. Generally, at 6 hours after plating, B2 neurons displayed a low-frequency spontaneous activity before the treatment (*n* = 8 in each group; Figure 6B). These cells strongly increased their firing activity after the washout of 20 μM serotonin applied for 10 minutes, switching their firing rates from 0.06 ± 0.06 spikes/s to 0.72 ± 0.14 spikes/s (vs. 0.12 ± 0.07 spikes/s in control group; *P* < 0.001, Bonferroni’s *post-hoc* test). No significant change was observed after the washout of high-KCl solution, thus confirming that washout completely depleted the potassium-induced activity. The same cells were tested again at 24-hour time-point and no spontaneous activity was detected in all the experimental groups. On the other hand, C1 neurons were always completely silent (Figure 6C).

These observations indicate that *Helix* isolated cells did not show any prolonged spontaneous activity triggered by 5-HT application, differently from polysynaptic circuits recorded on MEA devices (Figure 4).

In addition, we also investigated whether the activity of 5-HT-treated circuits may be ascribed to changes in cellular excitability by considering the firing rate of single cells induced by a depolarization pulse (+ 0.5 nA for 10 s). Isolated B2 neurons showed the same response in all experimental groups (Figure 6D). Interestingly, we observed a general increase in excitability from 6- to 24-hour time-points in C1 neurons (Figure 6E). A two-way ANOVA for repeated measures confirmed a significant effect of time (F_(2,25)_ = 34.39, *P* < 0.0001). Here, the application of serotonin induced a rapid and strong increase in firing rate that is statistically significant if compared to control and KCl-treated groups (5-HT: 3.300 ± 0.661 spikes/s, *n* = 11; vs. control: 0.357 ± 0.192 spikes/s, *n* = 7; vs. KCl: 0.110 ± 0.023 spikes/s, *n* = 10; *P* < 0.001, Bonferroni’s *post-hoc* test). Thus, we can infer that C1 neurons bear receptors responsive for serotonin that are involved in regulating their excitability without modifying their membrane potential, as observed in the homologous *Aplysia* metacerebral cell [20,21].

In all experimental groups, we observed no changes in other membrane properties, such as input resistance, threshold potential and resting potential (data not shown). We further evaluated the presence of alterations in action potential (AP) kinetics, measuring peak amplitude, AP half-width duration, AP rise time, AP decay time, after-hyperpolarization and rheobase. For each parameter, no difference was found between treated and control groups (data not shown).

### 5-HT and KCl treatments modulate neurite outgrowth in *Helix* circuitry

To better understand firing dynamics and functional connections inferred from MEA recordings, a parallel evaluation of neurite outgrowth and morphological network was performed. We measured the number of neurites interconnecting neurons within the same cluster (intra-cluster) and among the 4 different clusters the neurite density (inter-cluster) to evaluate neurite densities. Originally, data measured from C1 and B2 neuron have been analyzed separately: we decided to include these values in the same group since their growth rates were very similar to each other.

**Figure 7:**
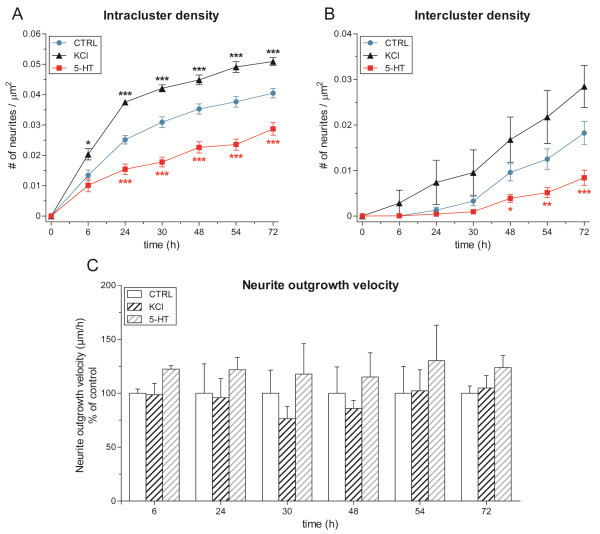
**Both KCl and 5-HT treatments affect *Helix *morphological connectivity.** (**A**) Bar graph of intra-cluster neurite density. We found that KCl application increased the number of neurites connecting the four cells of the same cluster. Conversely, a statistically significant reduction occurred after 5-HT treatment. Similar results were obtained considering the neurites projecting from one cluster to the other (inter-cluster density). **B**). While 5-HT induced an appreciable reduction, a less pronounced increase was detected in KCl-treated networks. (**C**) Bar graph of neurite outgrowth velocity showing that no growth defect was induced by the applied treatments.

As shown in Figure 7A, we found a general and rapid increase of intra-cluster neurite density with time reaching a limiting plateau value determined by the restricted surface area encompassed by each cluster (distance between two electrodes = 200 μm). This parameter is treatment-dependent. A two-way ANOVA for repeated measures revealed a significant effect of the treatment (F_(2,29)_ = 48.53, *P* < 0.0001) and time (F_(2,6)_ = 466.7, *P* < 0.0001) and a significant treatment by time interaction (F_(12,174)_ = 17.12, *P* < 0.0001). Particularly, we observed a statistically significant decrease of intra-cluster neurite density in 5-HT-treated circuits (*n* = 12) that started from 24 hours and persisted at the following time-points reaching the value of 0.029 ± 0.002 neurites/μm^2^*vs.* 0.040 ± 0.002 neurites/μm^2^ of control group (*n* = 12) at 72 hours (*P* < 0.001, Bonferroni’s *post-hoc* test). Conversely, the application of high-potassium solution induced an increase in neurite density with 0.051 ± 0.001 neurites/μm^2^ measured at 72 hours (*n* = 8; *P* < 0.001, Bonferroni’s *post-hoc* test).

We also considered the inter-cluster neurite density as an index of long-range neurites reflecting the putative connections among the four clusters (Figure 7B). During the first hours of development, none or few interconnecting neurites were observed confirming that signal propagation in the corresponding recordings was restricted to the cluster itself. Inter-cluster neurite density linearly increased from 24 to 72 hours with similar values in the KCl-treated and the control groups. The distance between clusters (600–1000 μm) guaranteed a continuous neurite extension without imposing a plateau value. We found that this enhancement was significantly impaired by the application of serotonin (i.e. at 72 hours: 0.008 ± 0.002 neurites/μm^2^ vs. 0.018 ± 0.003 neurites/μm^2^ of control group; *P* < 0.001, Bonferroni’s *post-hoc* test).

To directly assess the presence of a growth deficit induced by a single serotonin application, we measured the velocity of neurite elongation at the different time-points. Similar values were found in all experimental groups suggesting that the motility of growth cone has not been affected (Figure 7C). Thus, the lower neurite density may be ascribed to a direct effect of serotonin rather than a growth impairment, as observed in other models [22-24].

### *Helix* networks display functional connections during development

To investigate the overall circuit connectivity, we analyzed whether the synaptic connections detected in complex polysynaptic circuits of C1 and B2 neurons were formed according to previous electrophysiological characterizations of individual monosynaptic pairs [5] and whether the different treatments (i.e., KCl or 5-HT) modulate the functional connections, considering the evidence that neurite density is facilitated with KCl and depressed with 5-HT (cf., Figures 7A and 7B).

**Figure 8:**
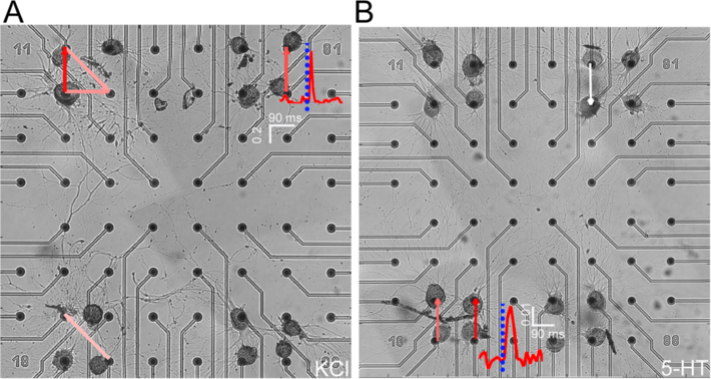
**Functional connectivity maps.** Examples of functional connectivity maps evaluated for the strongest 8 connections in a culture manipulated with (**A**) KCl or (**B**) 5-HT. The connection strength is coded by the color arrow: from red (strong) to white (weak). Inside the maps, two examples of cross-correlograms are reported. Scale bars: A: *x*-axis: 90 ms, *y*-axis: 0.2. B: *x*-axis: 90 ms, *y*-axis: 0.01.

**Figure 9:**
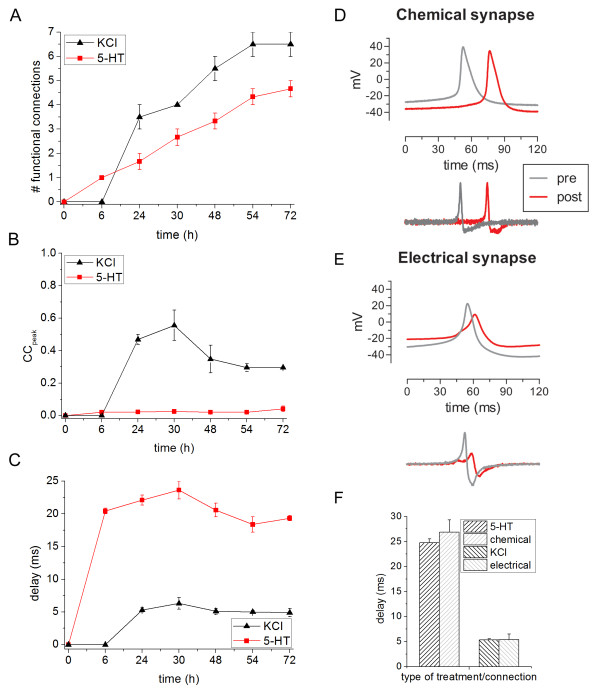
**Characterization of the functional connections during development.** (**A**) Number of detected functional connections in *Helix* neuronal cultures treated with KCl (black line) and 5-HT (red line). (**B**) Trend of the cross-correlogram peak values during development (KCl black, 5-HT red). (**C**) Estimation of the synaptic latency: in the case of 5-HT treatment, the delays are always higher than the corresponding with KCl. (**D**-**E**) Schematic representations of APs intracellularly recorded from pre and postsynaptic cells linked by a chemical (**A**) or electrical (**B**) connection. The first derivate of signals are shown in the bottom panels. (**F**) Bar graph showing the mean synaptic delays measured in relation to the type of connection (chemical and electrical) compared with the type of treatment (5-HT and KCl).

Figures 8 show two examples of functional connectivity (FC) maps relative to KCl (Figure 8A) and 5-HT (Figure 8B) treatments evaluated at the 48^th^ hour after plating. Two representative Cross-Correlations (CCs) were reported near the correspondent functional links: a relevant difference between KCl and 5-HT treatment on the cross-correlograms is the peak amplitude (about one order of magnitude; cf., also Figure 9 for a detailed characterization). FC maps were inferred from the CCs evaluated among all the active electrodes. Following the approach proposed in [25,26], we sorted all the statistically significant links based on the connection strengths, and we took into account only the 8 strongest links. In this way, it is likely we are considering the most reliable connections that would correspond to physical connections. From the FC analysis we did not find any relevant inter-cluster functional links even if we observed some long range connections in the morphological analysis (Figure 7B). The interaction measured by means of CC suggests that such distant neurons are not functionally connected. In fact, we found among inter-cluster electrodes: i) noisy and not significant CCs; ii) CC peaks falling into the bin centered in zero, thus representative of a random co-activation [26].

### 5-HT and KCl treatments modulate the synaptic strength and latency

As anticipated in the previous section, there is evidence that the two treatments modulate the FC of *Helix* neuronal networks. Thus, to characterize the functional links we estimated, for all the cultures and during their development, the strength (i.e., cross-correlogram peak value), and latency (i.e., the time difference between the occurrence of the cross-correlogram peak and zero).

Figure 9A shows the number of detected connections as a function of the network development, averaged over the number of cultures (i.e., 3 and 2 networks per 5-HT and KCl chemical treatment, respectively). All the detected connections were intra-cluster, since no inter-cluster links were found. Practically, this means that we were dealing with 12 and 8 not-functionally connected networks treated with 5-HT and KCl, respectively. KCl induces an increasing of the number of links compared with the networks manipulated with 5-HT showing similar results to that from measurement of neurite density (Figure 6A). After 54 hours in vitro, the networks treated with KCl reached a steady-state value of functional connections, while a linear monotonic trend was observed in the case of 5-HT. Interestingly, KCl-mediated connections were stronger than the 5-HT ones (after 6 hours in vitro). The trend of the estimated synaptic strength showed a peak at around the 30^th^ hours (0.56 ± 0.09), and converged towards an asymptotic value (0.30 ± 0.003). On the contrary, the estimated synaptic strength of the 5-HT networks was substantially flat with little oscillations and a small value around 0.03 (one order of magnitude smaller than KCl stimulation).

Next, we characterized the functional connections as latencies between the cross-correlated electrodes. KCl-treated networks presented a relatively fast synaptic transmission (about 5 ms) which remained rather constant during the development (Figure 9C). In the 5-HT treatment, the synaptic transmission was much slower: after 6 hours in vitro, the average latency was already at a plateau value of 20.4 ± 0.43 ms.

From the literature, it is known that *Helix* neurons may form both chemical and electrical synapses in culture [5]. Therefore, we consider the possibility that the two ranges of latencies measured may be related to the type of connection, more particularly to the synaptic delay. To sustain this hypothesis, we tested isolated C1-B2 and B2-B2 neuronal pairs for the presence of synaptic connections. Intracellular recording data of firing neurons were computationally transformed into the first derivative to reproduce extracellular signals acquired by MEA devices (Figure 9D and E). This manipulation is justified by previous modeling studies in which the neuron-microtransducer junction and the extracellular recorded waveform were mathematically modeled and characterized [27-29]. Then, we measured the delay time which occurred between two APs, one in the presynaptic cell and the other in the postsynaptic neuron triggered by the first signal (see Methods). We observed that cells electrically interconnected always fired in a synchronous fashion with a 1:1 relationship. Conversely, the action potentials recorded from chemically interconnected neurons were not always related to each other. In fact, an excitatory postsynaptic potential (EPSP) elicited by presynaptic firing may not be sufficient to trigger a new action potential in the postsynaptic neuron. For instance, EPSPs may not reach the threshold potential or, alternatively, may occur during the repolarization phase, when neuron is refractory to stimulation. These signals would result in membrane potential fluctuations that cannot be detected on MEA devices. Therefore, we decided to include in this analysis only spikes that are effectively correlated to each other. We found that chemical and electrical synapses are characterized by delays equal to 26.9 ± 2.5 ms and 5.4 ± 1.1 ms, respectively (Figure 9F). Such values are in agreement with the ones found at the network level (24.7 ± 0.8 ms and 5.3 ± 0.3 ms average values after 48 hours) when treated with 5-HT and KCl, respectively.

Taken together, these data suggested that KCl-treated *Helix* networks were prevalently interconnected through electrical synapses characterized by robust signals, synchronous firing and a strong coupling with short latency. On the other hand, polysynaptic circuits in which 5-HT has been repeatedly applied were formed by chemical connections, explaining a weaker connectivity and the longer latency. Therefore, we can infer that the enhancement of electrical versus chemical transmission in *Helix* circuits may be strongly up-regulated by means of KCl adding, and vice versa in the case of 5-HT.

## Discussion

In this work, we studied the electrophysiological activity and dynamics of neuronal ensembles made up of C1 and B2 *Helix* neurons during their in vitro development. The advantage of this biological preparation is the predetermined knowledge of cell type composition, biophysical properties and connectivity. In addition, by exploiting the MEA technology (i.e., extracellular recordings), we were able to perform long-lasting measures in order to characterize the development of *Helix* networks.

Here, the absence of spontaneous activity [10,30] was observed even during the formation of synaptically interconnected clusters, despite the increasing neuritic growth and arborization, and the establishment of synaptic connections. Taking into account this feature, we decided to trigger electrophysiological activity by applying two chemical treatments: KCl, to promote a general depolarization (unspecific stimulation); and 5-HT, to activate serotonergic receptors which are selectively expressed on B2 neurons. Following these stimulations, we observed similar dynamics during the development of these invertebrate circuits: the network firing early reached a value of firing rate and ISI, which remained almost unchanged during the development despite the connectivity maturation. This behavior is very peculiar, especially if compared with studies regarding the development of in vitro cortical neurons of rat embryos, [12,13], where it was found that cortical assemblies change their electrophysiological patterns as a function of the network maturation, perhaps because 1000-fold higher synapse density.

Although we observed that both KCl and 5-HT stimulations were sufficient to trigger firing, we found that only 5-HT efficiently induced a sustained long-lasting activity after the first application.

Serotonin has been frequently associated with changes in excitability. Brief 5-HT application has often been observed to induce immediate hyperexcitability of *Aplysia* sensory neurons, both in ganglion preparations [31-34] and in cell cultures [35,36]. Moreover, an increase in firing activity elicited by depolarizing current pulses has also been reported in rat cortical neurons after 5-HT exposure [37,38], suggesting a phylogenetically-conserved modulatory action for this neurotransmitter. Therefore, we evaluated the possibility that the observed long-term maintenance of firing activity could result from an increase of cell excitability. In addition to the B2 neuron response, a rapid increase of C1 neuron excitability was observed without alterations of other parameters, including membrane potential, input resistance and AP threshold. A similar effect was described in the *Aplysia* metacerebral cell [20,39], homologous to *Helix* C1 neuron. Even if the modulation of cell excitability may be a good explanation for triggering spontaneous activity in *Helix* circuits, it cannot be the only effector in promoting its maintenance. Behind the alteration of single cell properties, the emerging evidence would suggest changes in neuronal circuitry features as another putative cause, including the morphological and functional connectivity.

From a morphological point of view, our results showed an increase of neuritic density in cultures treated with KCl, while a reduction (compared to the non-stimulated networks) occurred in 5-HT stimulated networks. There is a large body of evidence that attribute to 5-HT an inhibitory role in the regulation of neurite outgrowth in invertebrate models [22-24], thus affecting both the development of arborizations and connectivity formation. In mammals, 5-HT has been implicated in shaping neuronal connectivity, e.g. decreasing neurite branching in rat cortical neurons during development [40] and impairing neurite density in mouse organotypic slice cultures [41].

From a functional point of view, *Helix* neurons may form both chemical and electrical connections in vitro[5,7]. Cross-correlation and latency analysis revealed a strong association between treatment and the type of circuitry connectivity. From the first hours of development, networks treated with 5-HT display synaptic latencies of about 20 ms, compared to the ones treated with KCl (about 5 ms after 24 hours). Comparing these values with those measured from interconnected pairs of neurons, we found a striking correspondence between MEA signal latency and the synaptic delays intracellularly recorded (Figure 9F).

The selective presence of the two types of synaptic connections may reflect the different strength of functional links estimated by means of cross-correlation analysis. The occurrence and the magnitude of chemical signal transduction vary dramatically depending on culture conditions, mainly related to the presence of trophic factors [5]. Moreover, phenomena such as neurotransmitter release modulation, synaptic delay, receptor desensitization, signal propagation along neurites and the postsynaptic refractory period may negatively affect the threshold potential to trigger an action potential in the postsynaptic cell. Conversely, the presence of gap junctions facilitate synchronous activity of coupled cells, allowing the direct transmission of electrical signal, thus explaining the 10-fold higher value of cross-correlogram peaks measured from KCl-treated cultures.

In support to our results, 5-HT has also been demonstrated to selectively prevent the formation of electrical synapses while allowing chemical synaptogenesis between identified *Helisoma* neurons [22,42]. The formation of electrical coupling between *Helisoma* neuronal pairs is thought to require an active growth state and neurite elongation [43-45], negatively modulated by 5-HT. However, a direct action of 5-HT on gap junctions has already been demonstrated in *Helisoma* neurons [46-48], as well as in developing rat cortical neurons [49].

On the other hand, the KCl-induced increase in neurite density may contribute to enhance cell-cell contact, thus promoting a higher coupling coefficient among cells [43]. Since gap junctions likely play a fundamental role in determining network synchronization [50,51], signals may reverberate among neurons until they return to a silent state by switching off the circuit.

Taken together, these observations suggest that the prevalence of chemical connections in 5-HT-treated circuits may be the cause of long-term maintenance of spontaneous activity. In this view, activity may be rapidly triggered by 5-HT application, through the depolarization of B2 neurons and the increased excitability of C1 neurons, and maintained by the continuous release of neurotransmitter from different firing neurons. Accordingly, repetitive applications of serotonin have previously been implicated in the enhancement of neurotransmitter release through presynaptic mechanisms [52-54].

## Conclusions

In this work, we characterized the dynamics exhibited by networks of *Helix* neurons coupled to MEAs. By exploiting the technology offered by these devices, we were able to study the development of the network and thus to appreciate possible changes in the network dynamics and firing patterns. In addition, we inferred the functional connections established among the neurons of the networks. Since spontaneously such invertebrate neurons are silent, we triggered their electrophysiological activity by delivering KCl or 5-HT. Under these treatments, we found that: i) KCl depolarizes the network for brief periods (minutes), without long-term effects. The dynamics of the network is fast (ISI_*peak*_ < 0.4 ms both for C1 and B2 cells during the entire development), and the detected functional connections show latencies compatible with electrical synapses (5.3 ± 0.3 ms average value after 48 hours). ii) 5-HT induces long-lasting firing activity that persists after the wash-out especially in B2 neurons. Compared to KCl-induced dynamics, we observed that 5-HT treated networks showed a slower dynamic (ISI_*peak*_ > 0.7 ms both for C1 and B2 cells during the entire development) and signal latencies characterized by delays of 24.7 ± 0.8 ms, suggesting that these circuits are mediated by chemical synapses. Finally, although the number of functional connections growth as far as the networks develop following a trend comparable with the neuritic outgrowth, we did not find statistically differences in the firing patterns during development as only little deviations-modulations are observed on a rather stable baseline activity.

## Methods

### Cell culture

Juvenile *Helix aspersa* land snails were purchased from local breeders. Cell cultures were performed as previously described [4]. Briefly, C1 and B2 neurons were isolated from cerebral and buccal ganglia, respectively, and grown under non-adhesive conditions overnight [5]. The day after, *Helix* somata were plated on poly-L-lysine (Sigma, Milano, Italy) pre-treated MEA devices or plastic dishes [10].

Four polysynaptic circuits (clusters) *per* MEA with a well-known composition (two clusters with 2 C1 + 2 B2, and 2 clusters with 4 B2, Figure 1A) were assembled taking into account the MEA electrode disposition. Each cell was plated on a single electrode to minimize signal dispersion. We defined as “*intra-cluster connectivity*” the functional links among the closest cells (i.e., with a Euclidean distance not greater than *d*·√2, where *d* = 200 μm is the distance between two electrodes), while as “*inter-cluster connectivity*” the functional connections among far electrodes (Figure 1A).

The same MEA device was recorded at 6-, 24-, 30-, 48-, 54- and 72 hours after plating.

To investigate the effect of 5-HT and high-KCl on synaptically-isolated cells, C1 and B2 somata were singularly plated, avoiding cell-to-cell contact, on plastic Petri dishes (Falcon #1006) coated with poly-L-lysine as adhesive substrate.

### Micro-Electrode Arrays, experimental setup and extracellular recordings

*Helix* neurons were plated over arrays (MEA 1060, Multi Channel Systems, Reutlingen, Germany) of 60 planar ITO (Indium Tin Oxide) microelectrodes (30 μm diameter, 200 μm spaced) and kept alive in healthy conditions up to 3 days. The experimental set-up is based on the MEA 60 system (Multi Channel Systems, MCS, Reutlingen, Germany): it includes a mounting support with integrated 60 channels, pre- and filter amplifier (gain 1200×), and a personal computer equipped with a PCI data acquisition board for real time signal monitoring and recording. The commercial MC_Rack software (MCS) was used for on-line visualization and raw data storage; data were further processed by using specifically developed software tools (cf., Data Analysis). Raw data were band pass filtered at 10 Hz to 3 kHz and sampled at 10 kHz per channel. Figure 1B and C display 1 second of electrophysiological activity coming from a C1 (Figure 1B) and a B2 (Figure 1C) neuron.

### Intracellular recordings

Electrophysiological parameters (e.g., membrane potential, input resistance, firing rate and action potential kinetics) were measured using conventional intracellular recordings.

To assess the presence of functional connections in *Helix* circuitry, intracellular techniques were coupled with MEA recordings in order to selectively stimulate one neuron with a supra-threshold current pulse and observe the propagation of signal among the other interconnected neurons.

To measure synaptic delays in *Helix* neurons chemically or electrically interconnected, C1-B2 or B2-B2 were paired in culture and electrophysiologically tested 48 hours after plating, as previously described [5]. After that the presence of an electrical or a chemical synapse was determined, cells were recorded during active firing (if necessary, triggered by a short depolarization pulse). Signal traces were then transformed into the first derivate by using GraphPad Prism version 5 (GraphPad Software, San Diego, CA) and the distance between the two related peaks were measured. In chemical synapses, the efficacy of EPSP to trigger an AP is extremely variable, so we defined as “efficacious EPSP” as depolarization event sufficient to reach the threshold potential and elicit a spike into the postsynaptic cell. We decided to include only this category of EPSPs in this analysis, since “not-efficacious EPSP” cannot be detected on MEA devices.

### Experimental protocol and dataset

All recordings started 10 min after positioning the MEAs upon the plate of the amplifier to let the cultures recover from the mechanical and thermal stress due to transfer from the incubator. Recordings were performed at room temperature.

### Spontaneous activity - control networks

Spontaneous activity was recorded for 30 min after a 10 min recovery period. We repeated such recording at the following time-points: 6, 24, 30, 48, 54, 72 hours after the plating. This protocol was applied to three networks composed of 4 C1 and 12 B2 neurons. The placement of the neurons over the MEA is illustrated in Figure 1A.

### Stimulation protocol

The activity of 5 cultures was recorded at the same time-points considered for the control networks. The complete stimulation protocol consisted of the following steps:

(1) Spontaneous activity was recorded for 30 min after a 10 min recovery period;

(2) Cultures were then stimulated by perfusion of 5-HT or KCl. In particular, 3 cultures were stimulated with 5-HT, and 2 with KCl. These networks present the same topological placement of the controls (Figure 1A). 5-HT was dissolved in L-15 Leibovitz culture medium to a final concentration of 20 μM; high potassium physiological salt solution was specifically modified to obtain final concentrations appropriate for *Helix* (20 mM NaCl, 65 mM KCl, 5 mM MgCl2, 7 mM CaCl2, 20 mM HEPES, pH 7.6; modified from [11]). After 10 minutes cells were rinsed with culture medium (7.5 ml/min) with 5 volumes of the recording chamber (1 volume = 2 ml). Flow was actively controlled by a peristaltic pump (Ismatec ISM829; Glattbrugg, Switzerland).

(3) Spontaneous activity was recorded for further 30 min after the stimulation.

### Data analysis

The characterization of the network dynamics exhibited by *Helix* cultures has been performed by computing the common first-order statistical algorithm, namely mean firing rate (MFR) and inter spike interval (ISI). Functional connectivity was inferred by the computation of the cross-correlation function among the recording sites. A brief description is furnished as follows:

### Spike detection and Instantaneous Firing Rate (IFR)

Spiking activity (Figure 1B and C) exhibited by *Helix* neurons has been detected by using a previously developed spike detection algorithm named ‘Precise Timing Spike Detection’ (PTSD) [55]. Briefly, such algorithm makes use of an independent threshold for each channel computed according to the standard deviation of the biological and thermal noise. For the presented experiments we set the spike detection parameters as follows: differential threshold (DT), 8 times the standard deviation of the noise; peak lifetime period (PLP) = 80 ms, refractory period (RP) = 100 ms. The peak lifetime and the refractory period are related to the duration of a spike and the minimum interval between two consecutive events.

The instantaneous firing rate (IFR) is computed by dividing the detected spikes in a small window of size Δ*t* by the bin width [56]. Such small window is realized by means of a Gaussian kernel of width equals to Δ*t* = 5 s. By dividing the total number of spikes for the entire recording duration, we can estimate the mean firing rate (MFR) of the network.

### Inter spike interval (ISI)

The probability density of time intervals between adjacent spikes is called the inter spike interval (ISI) distribution [57]. Such distribution has been computed by binning the spike train with a time window equal to 20 ms.

### Cross-Correlation and functional connectivity maps

Cross-Correlation (CC) function was built by considering the spike trains of two recording site [19]. The frequency at which a spike firing was recorded in one recording site (‘target site’) relative to the spike firing in another recording site (‘reference site’) as a function of time was measured and a CC function was evaluated considering all the pairs of spike trains. Mathematically, CC reduces to a simple probability *C*_*xy*_(*τ*) of observing a spike in a train *Y* at time (*t* + *τ*), because of a spike in another train *X* at time *t*; *τ* is called time shift or time lag; in this work it was set to 3 ms. Connection strength between the recording sites was evaluated on the basis of the peak value of the CC function, named *C*_*peak*_. Connection directionality was accounted from the sign of the corresponding peak latency. From the statistically relevant *C*_*peak*_, functional connectivity maps were estimated.

Functional connectivity (FC) captures patterns of deviations from statistical independence between distributed and often spatially remote neuronal units [58], measuring their correlation/covariance.

To estimate reliable FC map it is fundamental to threshold the connectivity matrix generated by the CC algorithm in order to consider only “true” and then strongest connections. A procedure to select the strongest functional links is necessary because a CC value (i.e., a *C*_*peak*_) is computed for each electrode pair independently of the existence of a direct or indirect (causal) link, a simply random co-activation or a noisy link. In this analysis, we estimate the FC maps by considering only the strongest links to avoid possible false positive connections and to focus on the most reliable connections (i.e., by considering a small number of links with respect to the total connections identified by the statistical method). In particular, we considered the 8 strongest [26].

### Morphological analysis

At each time-point, MEAs were observed on an Eclipse TE200 inverted microscope (Nikon Instruments, Tokyo, Japan) and images were acquired with a Monochrome Evolution QE camera (MediaCybernetics, Bethesda, MD). Two parameters of neurite outgrowth were assessed: neurite density and velocity of elongation.

With this aim, we considered boxes with fixed area equally distant between two neurons or two clusters of neurons, defined as intra-cluster or inter-cluster type, respectively. For each box, the total number of neurites virtually crossing the selected region were counted and values were divided by the surface area. We refer to this measurement as neurite density.

In order to quantify neurite elongation rates, we trace out 40 neurites from photographs taken sequentially for each MEA and neurite outgrowth was quantitatively measured using a semi-automated analysis system with Image Pro Plus version 6.3 (MediaCybernetics, Bethesda, MD). Neurites were selected for the presence of a growth cone actively growing. Growth velocity rates for each time point were calculated measuring the neurite extension as a function of time and expressed as the percentage of control values.

### Statistics

Data were expressed as means ± s.e.m. Statistical analysis was performed using GraphPad Prism version 5 (GraphPad Software, San Diego, CA). Statistical significance between group means was assessed using ANOVA analysis (one or two-way and with or without repeated measures where appropriate), followed by the Bonferroni’s *post-hoc* test, or by the non-parametric Kruskal-Wallis test, if the normality assumption was not verified (Kolmogorov-Smirnov normality test). Significance levels were set at *P* < 0.05.

## Abbreviations

MEA: Micro-Electrode Array; PTSD: Precise timing spike detection; IFR: Instantaneous firing rate; MFR: Mean firing rate; ISI: Inter spike interval; CC: Cross-correlation; FC: Functional connectivity; AP: Action potential; 5-HT: Serotonin.

## Authors’ contributions

PM and CNGG, designed and performed the experiments; PM and CNGG analyzed the data; MG, SM helped interpret the results, and contributed to the preparation of the manuscript. All the authors participated to the manuscript writing. All authors read and approved the final manuscript.

## Supplementary Material

Additional file 1 **Quasi-periodic behavior of C1 and B2 neurons when treated with 5-HT.** (A) Coefficient of variation (CV) of the ISI for C1 (red) and B2 (blue) neurons during development. The dotted gray line shows the threshold (set at 0.2) to individuate a quasi-periodic firing activity. (B) Firing activity of the C1 (red) and B2 (blue) neurons during development. The yellow ellipses mark those frequency values correspondent to a real periodic regime. Click here for file
