# Supplementary material for: Selective modulation of chemical and electrical synapses of Helix neuronal networks during in vitro development
Source: BMC Neurosci. 2013 Feb 25;14:22. doi: 10.1186/1471-2202-14-22 (PMC3626754; doi:10.1186/1471-2202-14-22)
Supplement: Additional file 1 — Quasi-periodic behavior of C1 and B2 neurons when treated with 5-HT. (A) Coefficient of variation (CV) of the ISI for C1 (red) and B2 (blue) neurons during development. The dotted gray line shows the threshold (set at 0.2) to individuate a quasi-periodic firing activity. (B) Firing activity of the C1 (red) and B2 (blue) neurons during development. The yellow ellipses mark those frequency values correspondent to a real periodic regime. [file 1471-2202-14-22-S1.docx]

**
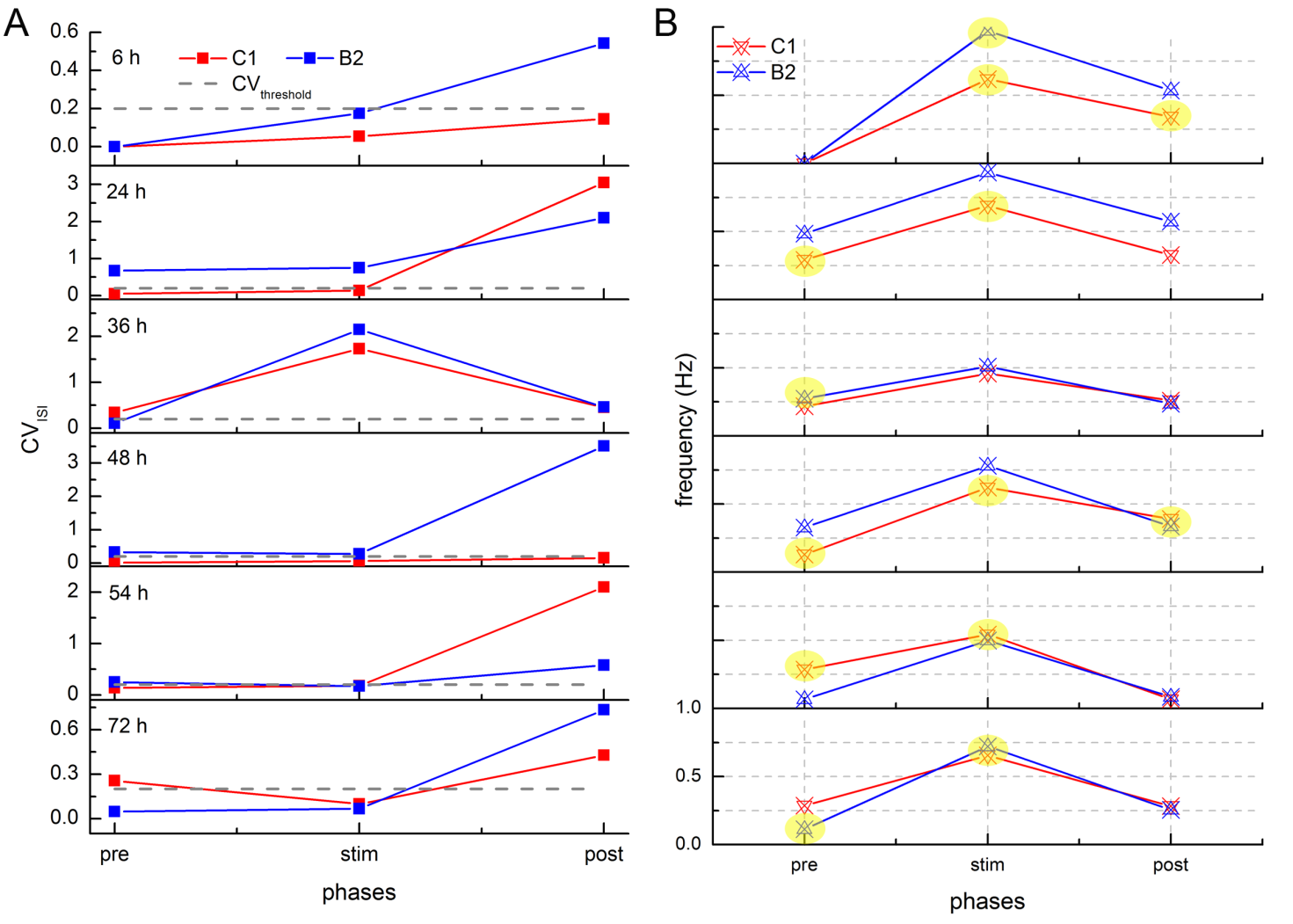
**

**Additional File 1. Quasi-periodic behavior of C1 and B2 neurons when treated with 5-HT**. (A) Coefficient of variation (CV) of the ISI for C1 (red) and B2 (blue) neurons during development. The dotted gray line shows the threshold (set at 0.2) to individuate a quasi-periodic firing activity. (B) Firing activity of the C1 (red) and B2 (blue) neurons during development. The yellow ellipses mark those frequency values correspondent to a real periodic regime.
